# Comparing Short Cognitive Screening Instruments in an Outreach Memory Clinic in Primary Care

**DOI:** 10.3390/ijerph22030410

**Published:** 2025-03-11

**Authors:** Rónán O’Caoimh, Sheena Cadoo, Brian Daly, D. William Molloy

**Affiliations:** 1 Health Research Board Clinical Research Facility, University College Cork, Mercy University Hospital, T12 WE28 Cork City, Ireland; 2 Centre for Gerontology and Rehabilitation, University College Cork, St Finbarr’s Hospital, Douglas Road, T12 XH60 Cork City, Ireland; brian.daly@nmdhb.govt.nz (B.D.); w.molloy@ucc.ie (D.W.M.); 3 Memory Resource Room, Mallow Primary Healthcare Centre, Mallow, P51 Y8EC County Cork, Ireland; sheena.cadoo@hse.ie; 4 Department of Pyschiartry, Nelson Hospital, Tipahi Street, Nelson 7010, New Zealand

**Keywords:** mental health, memory clinic, cognitive screening, primary healthcare, dementia, mild cognitive impairment

## Abstract

Few studies have investigated the possibility of offering outreach from hospital-based memory clinic services to primary care. Such models could potentially improve access to specialised mental healthcare. We report on the reliability and validity of a pilot cognitive screening pathway in general practice (GP) in Ireland. Consecutive patients with memory complaints attending two primary care clinics were screened and diagnosed clinically by a physician-run memory clinic in GP. Follow-up in secondary care confirmed the diagnosis. Inter-rater reliability (IRR) and diagnostic validity of the Quick Mild Cognitive Impairment (Q*mci*) screen was compared to the Montreal Cognitive Assessment (MoCA) and General Practitioner Assessment of Cognition (GPCOG). In all, 63 patients, 31 with subjective memory complaints (SMC), 16 with mild cognitive impairment and 16 with dementia were screened. Their median age was 73 and 67% were female. The IRR of the Q*mci* screen between GP and clinic was excellent (r = 0.89). The Q*mci* was more accurate than the GPCOG in identifying cognitive impairment; the area under the curve (AUC) was 0.95 versus 0.80 (*p* = 0.008). The Q*mci* and MoCA had similar accuracy, with an AUC of 0.95 versus 0.91 (*p* = 0.117), respectively, but was significantly shorter (*p* < 0.001), suggesting it may be a useful instrument in this setting. Based on these results, a definitive study is now planned to examine the benefits and challenges of utilizing these instruments as part of establishing an outreach memory clinic service in primary care.

## 1. Introduction

Dementia is a growing public health concern with epidemiological evidence showing that its prevalence at population level is continuing to increase worldwide [1,2]. Early identification of mild cognitive impairment (MCI), a prodromal stage characterised by cognitive symptoms but retained independence in daily activities [3], is particularly important in primary care to prevent or slow onset of dementia [4,5]. The impetus for this has increased with the recent development of disease modifying therapies for Alzheimer’s disease [6]. However, diagnosing and managing cognitive impairment including dementia and MCI in primary care, especially in this changing environment, remains challenging [4,7]. Barriers to diagnosis include time constraints in busy clinical general practice (GP), insufficient appointments and capacity, deficits in knowledge and experience and communication difficulties with secondary care [4,7,8,9]. There is also evidence that both patients and clinicians are reluctant to raise concerns in primary care [9]. Further, the lack of availability of sensitive and specific short cognitive screening instruments (CSIs) and insufficient evidence for screening or case finding programmes and pathways in this setting hamper timely identification of emerging cognitive decline in GP [6,8].

These mirror broader challenges delivering mental health services in the community in low- and middle-income [10] as well as higher-income countries [11]. Many countries have infrastructural, financial and management problems, which limit how they can provide these services to communities [10]. Four areas have been identified to better improve the provision of such services worldwide including reducing stigma, creating new models of mental health systems, enhancing research capacity, and implementing prevention programmes [12]. These are especially relevant for the diagnosis of MCI and dementia in GP, where brain health strategies and preventative guidance [13,14] could best be delivered to those with early cognitive symptoms [6,14]. At present, while many family physicians recognise their role in the diagnosis and management of cognitive decline, few engage patients in preventative measures [15]. Many of those working in primary care consider that healthcare systems do not facilitate this or support them to diagnose and management these patients [16]. In this model, most patients are referred to secondary care, to memory clinics which provide specialised, collaborative cognitive assessment and management services with streamlined post-diagnostic support pathways [17]. Reflecting these challenges, the development of cognitive impairment continues to be under-recognised in primary care [7,18,19] with at least half of all patients diagnosed elsewhere [20,21,22]. Early stages of cognitive decline such as MCI, as it often presents with more subtle cognitive changes, is particularly difficult to diagnose in GP [23,24]. In the United States for example, less than 10% of cases of MCI are diagnosed in GP with only 0.1% of primary care physicians and practices diagnosing at expected rates [24].

A specific barrier to the timely diagnosis of cognitive impairment in primary care is cognitive testing. The administration time of currently available instruments in primary care takes approximately 20 min [25], with marked constraints on more complex and detailed neuropsychological testing, which hence, are rarely employed. This necessitates the utilisation of short CSIs and informant questionnaires in GP [26]. Features of acceptable CSIs for use in primary care include brevity, ease of administration and adequate sensitivity and specificity to differentiate between different stages of cognitive decline [27]. Although several CSIs are available for use in primary care [26,27,28,29], including those designed specifically for use in GP, the most commonly used tests remain the abbreviated mental test score and the Mini-Mental State Examination (MMSE) [30]. The MMSE, while short and useful in separating dementia from normal cognition, is insensitive to early cognitive changes and has poor accuracy in detecting MCI; in a recent systematic review, it was found to have only 73% (pooled) sensitivity, albeit with higher (83%) specificity [26]. The GPCOG [31] is one of the few CSIs designed and recommended for use in primary care [26,28]. It is shown to have similar or better diagnostic accuracy compared to the MMSE in detecting MCI but has a shorter administration time of <4 min [26,31]. While the MoCA is widely used in multiple settings and has excellent overall diagnostic accuracy for MCI and dementia, albeit with lower specificity [32,33], it is not widely validated in GP due to the need for mandated online training (certification) and relatively long administration times up to 15 mins [33]. It is also shown to have high false positive rates in primary care with one recent study in France finding that 62% of adults ≥50 years had MCI using the MoCA [33]. On the other hand, the GPCOG was designed specifically for use by GPs, is short to administer and is deemed acceptable by clinicians and patients [31]. It has not, however, been validated widely to detect MCI specifically [34], only dementia and more broadly, cognitive impairment [35,36]. A recent working group advised that additional tools that meet the needs of primary care are required and that these should be benchmarked against gold standard assessment [27].

While community-level screening for cognitive impairment is not recommended [37,38] and few screening programmes have been trialled in GP [19], some recent studies have highlighted that pathways starting in primary care and linking into secondary care are feasible [39]. Although memory clinics, usually based in hospitals and secondary care, have traditionally provided diagnostic capabilities and instigated management strategies, these remain a costly and limited resource with considerable heterogeneity across services and are often inaccessible to community-dwellers, especially in more rural communities [40,41]. A ‘hybrid’ ‘remote’ or ‘mobile’ outreach model incorporating the expertise and dedicated resources of a memory clinic with the convenience and acceptability of primary care for patients, especially older adults and those in rural communities, is appealing [42]. Such outreach models can streamline assessment, improve access and provide early detection and diagnostic rates [43]. Examples include those in rural provinces in Canada where a “one stop” review incorporating a clinical assessment and telehealth follow-up is provided [42]. It is currently not known how best to optimize cognitive testing in these outreach clinics or, in particular, which short CSI is best. In Australia, which has similarly remote rural communities with limited access to secondary care, screening pathways in GP with onward referral to secondary care has been trialled [43]. As in many other settings, the MMSE is widely used in studies examining these models [42,43], though concerns over poor sensitivity in those with early cognitive impairment or false positives or negatives potentially causing patient distress and reduced rates of onward referral, respectively, have been highlighted [43]. Hence, there is a need for more research to better examine short CSIs in this setting, particularly instruments that have more accuracy in MCI.

The objective of this pilot project, which is a secondary analysis of a larger study of a memory clinic in Ireland [44], was to explore the diagnostic accuracy of three different short CSIs (conducted as part of a community-based memory clinic screening initiative supported by secondary care with an onward referral pathway to confirm the diagnosis in a formal memory clinic). The CSIs to be examined include the GPCOG, MoCA and the more-recently validated Quick Mild Cognitive Impairment (Q*mci*) screen [44,45], which while shown to have better accuracy compared to the MMSE and MoCA and is yet to be validated in primary care.

## 2. Materials and Methods

### 2.1. Sample

Consecutive middle aged and older adults aged ≥55 years of age attending primary care in two large GPs, one urban and one rural/suburban in County Cork, Ireland, over an approximately two-month period in 2014 were included. Patients were excluded if they were <55, had active depression or were non-English literate. Those with atypical dementia syndromes such as Parkinson’s disease and Lewy body dementia were also excluded as these typically present with exaggerated functional deficits and a different MCI syndrome [46]. Based upon the original validation of the Q*mci* screen against the Standardised MMSE [45], we calculated that a sample of 76 paired observations would be required to detect a 20% difference in sensitivity and specificity between the two tests at a significance level of 0.05 and power of 80%. All patients underwent a comprehensive clinical assessment including evaluation of baseline demographics (age, gender, number of years in education). Informant screening was performed using the Informant Questionnaire on Cognitive Decline in the Elderly-Short Form (IQCODE-SF) [47] and the eight-item Ascertain Dementia 8 (AD8) screening interview [48]. Based upon this, patients were diagnosed by a physician with either dementia, MCI or subjective memory complaints (SMCs) but normal cognition. Dementia was diagnosed using DSM-IV criteria [49]. A diagnosis of MCI was based upon a history of subjective and corroborated memory loss, without loss of social or occupational function with identifiable cognitive deficits per Petersen’s criteria [50] as refined by the National Institute on Aging-Alzheimer’s Association workgroup in 2011 [51]. SMCs were defined by a response to the question “how is your memory?” Those scoring ‘fair’ and ‘poor’ on a five-point Likert scale were classified as having SMCs provided they had no objective cognitive deficits on testing and no functional decline on questioning [52]. Active depression was screened for using the 15-point Geriatric Depression Scale [53], with those scoring 7 or more points excluded. All patients provided informed written consent. Ethical approval for the study was obtained in advance from the Clinical Ethics Committee of Cork University Hospitals (reference number: ECM 3 kkk 03/12/13).

### 2.2. Data Collection

An “outreach” memory clinic was created for the purposes of this pilot study as a proof of concept, though the data collection was conducted as part of a larger study of memory clinic pathways and CSI development [44]. The Q*mci* screen, MoCA and GPCOG were administered to each patient sequentially, but randomly by trained raters (Occupational Therapist and or General Practitioner) to reduce learning effects, who were blind to the final diagnosis of cognitive impairment. All patients were subsequently reviewed by a consultant Geriatrician with expertise in the diagnosis of dementia in a specialised memory clinic in a university hospital. A GP recruited all patients and provided details to a trained physician, who performed an ‘outreach’ function in each of the two primary care practices. These community-based memory clinics were held on average once per two weeks. This physician in turn conducted more detailed analysis and once a diagnosis was made referred patients to the university hospital memory clinic for confirmation with the goal that all patients would be seen within one month; the professor leading that unit was blind to the analysis that had been completed in the GP. The evaluation in secondary care has been described elsewhere [44] but in summary, the assessment was MDT-led and included detailed brain imaging (CT, MRI or PET), neuropsychological evaluation with a battery of assessments, and biomarker testing if required, to support the diagnosis.

### 2.3. Measures

The GPCOG was designed specifically for use in primary care, and takes approximately 5–6 min to complete [31]. It is composed of two steps, an observer-rated (Step 1, the GPCOG Informant) and neuropsychological testing (Step 2, the GPCOG Examination) component. There is separate scoring for each part. In Step 1, there are six questions (sentence registration, orientation, clock drawing, information on current events and sentence recall) with a total of 9 points available with higher points indicating better cognition. Where the patient scores 9/9, no significant cognitive impairment is suspected and no additional testing is required. Scores of 5–8/9 indicate that more information is required and the rater should proceed to Step 2. If the patient scores 0–4/9, cognitive impairment is suggested. In Step 2, six questions are asked by an informant detailing how the patient compared 5–10 years prior. If the patient scores 0–3, cognitive impairment is suspected and more detailed assessment is indicated. This has reported age and educational bias [54]. The Q*mci* screen is a short CSI incorporating six subtests across five domains including orientation, registration, clock drawing, delayed recall, verbal fluency (animal naming) and logical memory (a test of immediate verbal recall of a short story), scored out of 100 points with an administration time of approximately 5 min [55]. It is correlated with more detailed neuropsychological testing including the Clinical Dementia Rating scale [56]. A score less than 62 suggests the presence of cognitive impairment, and less than 50, dementia [57]. Age and educational cut-off scores are available [57]. It has been validated as part of a community screening programme in Portugal [58]. The MoCA is a short CSI with usually high sensitivity for cognitive impairment, particularly MCI, over and above the MMSE; it is composed of five main cognitive domains covering memory, language, visuospatial, attention and cognitive control, and is scored out of 30 points with higher points implying better cognition an administration time of approximately 10–12 min [32].

### 2.4. Analysis

Analyses were conducted using SPSS 24.0 for Mac (SPSS, Inc., Chicago, IL, USA). The Shapiro–Wilk test was used to test normality and found that most data were non-normally distributed and hence non-parametric statistical approaches were taken. The Mann–Whitney U test and the Kruskal–Wallis test were used to compare any differences between continuous variables, while Chi Squared testing was used to examine differences between categorical variables. Spearman’s Rank Correlation Co-efficient was used to measure inter-rater reliability (IRR). Diagnostic accuracy of the three short CSIs was analysed using receiver operating characteristic curve analysis, taking the area under the ROC curves (AUCs). These were compared statistically using the DeLong technique [59].

### 2.5. Inter-Rater Reliability Testing

IRR testing was performed throughout the study for the Q*mci* screen. GPs were trained prior to the study on how to score the Q*mci* screen. Training involved a lecture on the use of the CSIs. The Q*mci* screen has been filmed for training and teaching purposes and was available to all raters as the instrument was newly developed at the time. IRR, between the GP and Memory clinic, was shown by comparing the results of the Q*mci* screen score obtained in the GP with a repeat in the memory clinic (*n* = 57). Six patients were assessed in the university memory clinic more than one month after the initial scoring by the GP and were therefore not included in the IRR analysis.

## 3. Results

In all, 63 patients were screened. This included 31 patients with SMC, 16 with MCI and 16 with dementia. The characteristics of these patients are presented in Table 1. The median age of all patients was 73 years, with an interquartile range (IQR) ± 17 years. Overall, 66.6% were female and the median number of years in education was 11 (IQR ± 4). The median Standardised MMSE score for the total sample was 27/30 (IQR ± 4), reflecting a high level of independence overall and the median Barthel Index score was 20/20. Patients with SMCs were significantly younger than those with MCI or dementia (*p* < 0.001). There was no statistically significant difference in number of years in education between individuals in the three diagnostic categories (*p* = 0.522). The median Q*mci* screen, MoCA and GPCOG scores were 60/100 (±24), 22/30 (±8) and 8/9 (±5), respectively. IRR testing between primary care and the clinic assessment was only performed for the Q*mci* screen and was excellent (*r* = 0.89).

**Figure 1:**
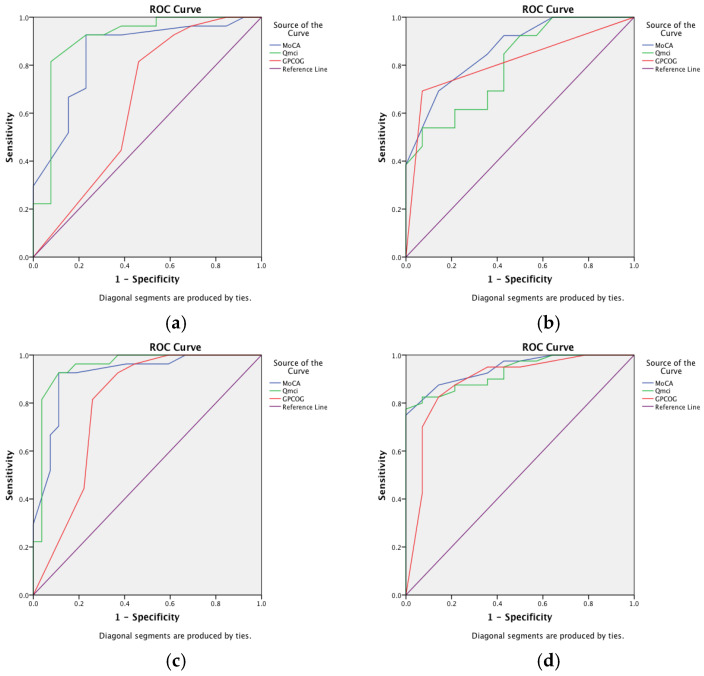
Receiver operating characteristic curves demonstrating the accuracy of the Quick Mild Cognitive Impairment (Q*mci*) screen and Montreal Cognitive Assessment (MoCA) and the General Practitioner Assessment of Cognition (GPCOG) in differentiating patients attending general practice with (**a**) mild cognitive impairment (MCI) from subjective memory complaints (SMCs) but normal cognition, (**b**) MCI and dementia, (**c**) SMC from cognitive impairment (MCI and dementia) and (**d**) dementia from everything else.

Figure 1 shows ROC curves comparing the Q*mci* screen, MoCA and GPCOG in their ability (diagnostic accuracy) to differentiate those with SMC but normal cognition from MCI and dementia. Their corresponding AUC values are presented in Table 2. The Q*mci* screen was more accurate than the GPCOG in differentiating MCI from SMC, with an AUC of 0.91 versus 0.67 (*p* = 0.01), respectively. There was no statistically significant difference in the ability of the Q*mci* screen and MoCA (AUC of 0.84) to differentiate MCI from SMC (*p* = 0.19). The Q*mci* screen had similar accuracy in differentiating those with SMC from those with cognitive impairment (i.e., MCI or dementia), with an AUC of 0.95 versus 0.91 (*p* = 0.117). Both the Q*mci* screen (*p* = 0.008) and MoCA (*p* = 0.02) were more accurate than the GPCOG (AUC of 0.80). All three CSIs had similar accuracy in separating out patients with dementia from MCI and or SMC but with normal cognition.

The GPCOG classified 34% of patients as having cognitive impairment (including 93% of those with dementia) with only 3.6% of patients with SMC but normal cognition misclassified. The median administration time for the Q*mci* screen was 4.4 (±1) minutes versus 8.2 (±2.5) for the MoCA and 2.5 (±1) minutes for the GPCOG. The administration time of the MoCA was statistically significantly (borderline) longer among those with greater cognitive impairment (*p* = 0.046). The Q*mci* screen had a statistically shorter administration time than the MoCA (*p* < 0.001). Table 3 presents the sensitivity and specificity for each of the three CSIs with optimal cut-offs based upon Youden’s Index [60]. Here, the optimal cut-off for cognitive impairment (MCI or dementia) was <20/30 for the MoCA, which yielded a sensitivity of 81% and specificity of 93%. The optimal cut-off for the Q*mci* screen was close to its published cut-off (<62) [44] at <60/100, which gave a sensitivity of 91% and specificity of 90%. The cut-off for the GPCOG for identifying cognitive impairment was <5/9; it had reasonable sensitivity at 64% but excellent specificity of 93%. At a higher cut-off (previously published <7/9 [36]), sensitivity was 75% and specificity 82%. For separating MCI from SMC, the optimal cut-off was <21/30 for the MoCA (sensitivity of 73% and specificity of 87%.) At its optimal cut-off for MCI (<26/30) [32], the MoCA had a sensitivity of 100% but a low specificity of just 33%. The optimal cut-off for the Q*mci* screen for MCI was <61/100, which gave a sensitivity of 94% and specificity of 81%. The GPCOG had a cut-off of <7/9 providing a sensitivity of 57% and specificity of 82%. The GPCOG was most accurate for identifying dementia with a sensitivity of 86% and specificity of 81%.

## 4. Discussion

This study presents a comparison between three short CSIs in a primary care population participating in a pilot outreach memory clinic in GP and shows that this screening pathway from primary care is reliable and accurate. The specific analysis shows that the Q*mci* screen and MoCA were statistically significantly more accurate that the GPCOG in differentiating patients presenting to GP with cognitive impairment (MCI and dementia) as well as being more accurate in separating MCI from SMC but normal cognition. Although the GPCOG was shorter to administer, it was less accurate, while the Q*mci* screen was longer but statistically quicker to score the MoCA. The GPCOG had particularly poor diagnostic accuracy with an AUC of 0.67 for differentiating those with MCI from those with SMC. All CSIs had short administration times, under 10 min with the GPCOG and Q*mci* screen having median times <5 min. The Q*mci* had excellent IRR in this analysis.

This study provides further evidence that the Q*mci* screen is an accurate instrument in identifying impairment across the cognitive spectrum [61] and confirms that it has comparable accuracy but is shorter than the MoCA [44]. It also re-affirms that the optimal cut-off for the Q*mci* screen for cognitive impairment is approximately <60/100 and for dementia, it is <50/100 across different populations and countries [62]. It likewise shows again that the optimal cut-off for the MoCA is lower than that published for the original validation cohort (<26/30) and instead is closer to <21/30, which was found in this study, albeit acknowledging that cut-off scores vary depending on the sample [63]. In this study, one of the first to specifically examine its ability to separate SMC from MCI, the GPCOG showed reasonable accuracy for cognitive impairment (AUC of 0.82) but generally low sensitivity (64%) at a cut-off of 5/9. In this and other studies based in primary care, the GPCOG has shown better sensitivity (89%) but relatively lower specificity of 61% at a higher cut-off of 7/8 of 9 [35,36].

In addition to comparing the accuracy of short CSIs in a community sample in GP and the reliability of screening with the Q*mci* screen compared to clinic practice in secondary care, this study validates these instruments in a new setting, that of the emerging models of community-based memory clinics run in conjunction with primary care [43]. Indeed, such models have utilised the GPCOG to determine who is referred on to secondary care for further assessment and management [43]. A similar pathway in Australia found that 68.9% met criteria for onward referral [43]. A similar percentage merited further testing in another study in India [64]. In this study in Ireland, half were formally diagnosed with cognitive impairment (MCI or dementia). At the heart of such models is the need to educate the workforce to improve confidence with screening and assessment [43]. Hence, this study supports evidence from recent working groups, which have highlighted that while mass screening for cognitive impairment is not recommended, clear integrated pathways for persons with symptoms presenting to primary care should be identified and developed [8]. It also supports previous calls to further validate these short CSIs in primary care against gold standard pathways [27], i.e., in secondary care, more detailed assessments are performed by specialists trained in diagnosing and managing cognitive impairment. This will require adequate resourcing in terms of education, infrastructure and equipment, but will improve patient diagnostic rates, patient experience and ultimately improve outcomes [8].

This study has a number of strengths and limitations. Strengths of the study include the relatively detailed assessment of cognitive impairment that was conducted. Limitations include the small number of patients included, underpowering the study to show superiority of one instrument over another and limiting this study to a non-inferiority study. The sequence of assessments using the different short CSIs could have introduced habituation and learning effects, albeit these were delivered in a random sequence to reduce this. IRR testing was only performed for the Q*mci* screen limiting the utility of reliability testing. Further, normal controls were not included, rather those with SMCs, which is not a benign condition and can predict future risk of dementia [65], potentially introducing spectrum bias. Another limitation is the risk of demographic bias, given the homogenous sample (Caucasian Irish middle-aged and older adults), reducing generalisability. The study dates to 2014, further reducing generalisability. This reflects that the study is a secondary analysis of an existing database (gathered approximately ten years ago), which is being reassessed to gather evidence to support further study of this pathway, given the limited evidence in the literature to date.

## 5. Conclusions

This pilot study shows the validity of short CSIs in an outreach primary care clinic pathway. It highlights that the MoCA and Q*mci* screen are more accurate, albeit have longer administration times than the GPCOG. Though evidence for routine cognitive screening in primary care is lacking, consensus suggests that early recognition of symptomatic individuals can benefit patients, relatives and mental health services alike [66]. Due to time and other constraints, accurate and short CSIs, which are simple to administer, are needed in primary care. The Q*mci* screen had excellent IRR and was shorter than the MoCA and more accurate in differentiating MCI from normal cognition and dementia than the GPCOG, suggesting that it should be studied in larger studies in GP to investigate if it strikes the best balance between administration time and diagnostic accuracy and hence if it could be an ideal brief screen for use in GP. Additional studies with an adequately larger sample size are now required to confirm these findings and further study of similar pathways to show their benefits and risks is important.

## Figures and Tables

**Table 1:** Characteristics of patients reviewed in general practice comparing those with subjective memory complaints (SMCs) but normal cognition to those with mild cognitive impairment (MCI) and dementia.

Diagnosis	Total (*n* = 63)	SMC (*n* = 32)	MCI (*n* = 16)	Dementia (*n* = 16)	*p* = x
Age in years (Median and IQR)	73 (81–64 = ±17)	66 (74–59 = ±15)	74.5 (81–69 = ±12)	79.5 (83–75 = ±8)	X^2^ = 16 (df = 2) <0.001
Education (Median and IQR)	11 (14–10 = ±4)	12 (15–11 = ±4)	11 (14–10 = ±4)	12 (13–11 = ±2)	X^2^ = 1.3 (df = 2) 0.522
Sex (% Female)	67%	61%	69%	75%	X^2^ = 0.93 (df = 2) 0.693
IQCODE (Median and IQR)	3.9 (4.3–3.5 = ±0.8)	3.16 (3.7–3 = ±0.7)	3.7 (4–3.6 = ±0.4)	4.3 (4.7–4 = ±0.7)	X^2^ = 12 (df = 2) 0.002
AD8 (Median and IQR)	5 (7–4 = ±3)	2 (5–0 = ±5)	4 (5–3 = ±2)	7 (8–6 = ±2)	X^2^ = 17 (df = 2) <0.001
SMMSE (Median and IQR)	27 (29–25 = ±4)	29 (30–28 = ±2)	26.5 (28–25 = ±3)	20.5 (24–18 = ±6)	X^2^ = 36 (df = 2) <0.001
Q*mci* screen (Median and IQR)	60 (71–47 = ±24)	71 (74–59 = ±15)	54.5 (59–43 = ±16)	40.5 (48–32 = ±16)	X^2^ = 40 (df = 2) <0.001
Q*mci* admin time (Median and IQR)	4.4 (5–4 = ±1)	4.0 (4–4 = 0)	4.4 (5–4 = ±1)	4.84 (5–4 = ±1)	X^2^ = 4 (df = 2) 0.144
MoCA (Median and IQR)	22 (25–17 = ±8)	25 (27–22 = ±5)	18 (22–17.5 = ±4.5)	14.5 (17–11 = ±6)	X^2^ = 35 (df = 2) <0.001
MoCA admin time (Median and IQR)	8.2 (10–7.5 = ±2.5)	7.7 (8–6.5 = ±1.5)	8.5 (9–8 = ±1)	10 (10–10 = ±0)	X^2^ = 6 (df = 2) 0.046
GPCOG (Part 1: Examination) (Median and IQR)	8 (9–4 = ±5)	8 (9–8 = ±1)	7 (9–4 = ±5)	2.5 (4–1 = ±3)	X^2^ = 22 (df = 2) <0.001
GPCOG (Part 2: Informant) (Median and IQR)	4 (6–2 = ±4)	6 (6–4 = ±2)	4 (4–3 = ±1)	2 (3–0 = ±3)	X^2^ = 32 (df = 2) <0.001
GPCOG admin time (Median and IQR)	2.5 (3.4–2.4 = ±1)	2.4 (2.8–2.2 = ±0.6)	2.5 (3–2.5 = ±0.5)	3 (3–2.5 = ±0.5)	X^2^ = 1 (df = 2) 0.468
GPCOG (% Cognitive impairment)	34%	3.6%	36%	93%	X^2^ = 33 (df = 2) <0.001

AD8 = Ascertain Dementia 8 screening interview; GPCOG = General Practitioner Assessment of Cognition; IQCODE = Informant Questionnaire on Cognitive Decline in the Elderly; MoCA = Montreal Cognitive Assessment; Q*mci* = Quick Mild Cognitive Impairment screen; SMMSE = Standardised Mini-Mental State Examination.

**Table 2:** Comparison of the diagnostic accuracy of the Quick Mild Cognitive Impairment (Q*mci*) screen, Montreal Cognitive Assessment (MoCA) and the General Practitioner Assessment of Cognition (GPCOG) in differentiating patients attending general practice with (a) mild cognitive impairment (MCI) from subjective memory complaints (SMCs) but normal cognition, (b) MCI and dementia, (c) SMC from cognitive impairment (MCI and dementia) and (d) dementia from everything else.

Corresponding Figure	Comparison *	MoCA	Q*mci* Screen	GPCOG
		Area Under the Curve (95% Confidence Intervals)		
a	MCI versus SMC	0.84 (0.72–0.98)	0.91 (0.79–1.0)	0.67 (0.44–0.85)
	MoCA vs. Q*mci*; *p* = 0.19 MoCA vs. GPCOG; *p* = 0.02 * Q*mci* vs. GPCOG; *p* = 0.01 *			
b	MCI versus Dementia	0.87 (0.73–1.0)	0.80 (0.63–0.97)	0.81 (0.64–0.99)
	MoCA vs. Q*mci*; *p* = 0.364 MoCA vs. GPCOG; *p* = 0.397 Q*mci* vs. GPCOG; *p* = 0.491			
c	Cognitive Impairment (MCI and dementia) versus SMC	0.91 (0.84–1.0)	0.95 (0.89–1.0)	0.82 (0.69–0.94)
	MoCA vs. Q*mci*; *p* = 0.117 MoCA vs. GPCOG; *p* = 0.02 * Q*mci* vs. GPCOG; *p* = 0.008 *			
d	Dementia versus MCI and SMC	0.94 (0.89–1.0)	0.93 (0.86–1.0)	0. 91 (0.82–1.0)
	MoCA vs. Q*mci*; *p* = 0.910 MoCA vs. GPCOG; *p* = 0.271 Q*mci* vs. GPCOG; *p* = 0.614			

* Statistically Significant.

**Table 3:** Sensitivity and specificity with optimal cut-off scores for cognitive impairment, comparing the Quick Mild Cognitive Impairment (Q*mci*) screen, Montreal Cognitive Assessment (MoCA) and the General Practitioner Assessment of Cognition (GPCOG) in differentiating patients attending general practice with (a) mild cognitive impairment (MCI) from subjective memory complaints (SMCs) but normal cognition, (b) MCI and dementia, (c) SMC from cognitive impairment (MCI and dementia) and (d) dementia from everything else.

Instrument	MoCA			Q*mci* Screen			GPCOG		
Diagnostic Category	Youden’s Index (Optimal Cut-Off)	Sensitivity	Specificity	Youden’s Index (Optimal Cut-Off)	Sensitivity	Specificity	Youden’s Index (Optimal Cut-Off)	Sensitivity	Specificity
MCI versus SMC	<21/30	0.73	0.87	<61/100	0.94	0.81	<7/9	0.57	0.82
MCI versus Dementia	<17/30	0.81	0.73	<53/100	0.86	0.56	<3/9	0.64	0.86
MCI and Dementia versus SMC	<20/30	0.81	0.93	<60/100	0.91	0.90	<5/9	0.64	0.93
Dementia versus SMC and MCI	<18/30	0.94	0.78	<55/100	0.94	0.79	<5/9	0.86	0.81

## Data Availability

Data may be accessed upon request to the corresponding author.
